# Analysis of Pain and Analgesia Protocols in Acute Cerulein-Induced Pancreatitis in Male C57BL/6 Mice

**DOI:** 10.3389/fphys.2021.744638

**Published:** 2021-11-22

**Authors:** Mattea Durst, Theresia Reding Graf, Rolf Graf, Mareike Kron, Margarete Arras, Dietmar Zechner, Rupert Palme, Steven R. Talbot, Paulin Jirkof

**Affiliations:** ^1^Centre for Surgical Research, University Hospital Zurich, University of Zurich, Zurich, Switzerland; ^2^Pancreas Research Laboratory, Department of Visceral Surgery & Transplantation, University Hospital Zurich, Zurich, Switzerland; ^3^Rudolf-Zenker-Institute of Experimental Surgery, University Medical Center, Rostock, Germany; ^4^Unit of Physiology, Pathophysiology and Experimental Endocrinology, Department of Biomedical Sciences, University of Veterinary Medicine, Vienna, Austria; ^5^Institute for Laboratory Animal Science, Hannover Medical School, Hanover, Germany; ^6^Office for Animal Welfare & 3R, University of Zurich, Zurich, Switzerland

**Keywords:** mouse model, pain, analgesia, cerulein-induced acute pancreatitis, C57BL/6 Mice

## Abstract

Pancreatitis is known to be painful in humans and companion animals. However, the extent of pain in experimental mouse models of acute pancreatitis is unknown. Consequently, the severity classification of acute pancreatitis in mice is controversially discussed and standardized pain management is missing. In this study, we investigated acute Cerulein-induced pancreatitis with pain-specific and well-being orientated parameters to detect its impact on mice. Male C57BL/6J male mice were injected with Cerulein; animals that received saline injections served as control group. The animals were observed for weight change and water intake. To assess pain, behaviors like stretch-and-press and reduced rearing, the Mouse Grimace Scale, and von Frey hypersensitivity were assessed. Fecal corticosterone metabolites and burrowing behavior were assessed to detect changes in the animal’s well-being. Pancreatitis severity was evaluated with amylase and lipase in the blood and pancreas histology. To investigate whether different analgesics can alleviate signs of pain, and if they influence pancreas inflammation, animals received Buprenorphine, Paracetamol in combination with Tramadol, or Metamizole in the drinking water. The calculated intake of these analgesics *via* drinking reached values stated to be efficient for pain alleviation. While pancreatitis did not seem to be painful, we detected acute pain from Cerulein injections that could not be alleviated by analgesics. The number of inflammatory cells in the pancreas did not differ with the analgesic administered. In conclusion: (1) Cerulein injections appear to be acutely painful but pain could not be alleviated by the tested analgesics, (2) acute pancreatitis induced by our protocol did not induce obvious signs of pain, (3) analgesic substances had no detectable influence on inflammation. Nevertheless, protocols inducing more severe or even chronic pancreatitis might evoke more pain and analgesic treatment might become imperative. Considering our results, we recommend the use of Buprenorphine *via* drinking water in these protocols. Further studies to search for efficient analgesics that can alleviate the acute pain induced by Cerulein injections are needed.

## Introduction

Acute pancreatitis in humans is a severe, life-threatening disease. To understand the mechanisms and pathogenesis of the disease on a cellular as well as a systemic level, and to analyze potential therapeutic interventions, researchers often rely on mouse models (Gorelick and Lerch, 2017; Silva-Vaz et al., 2019). While several approaches are available to induce inflammation of the pancreas in animals, Cerulein, a hormone analogous to cholecystokinin, which stimulates the secretion of pancreatic enzymes, is most commonly used in mouse models (Hyun and Lee, 2014). The model is simple, cheap, easy to apply, and reproducible (Gorelick and Lerch, 2017; Silva-Vaz et al., 2019). In 2018 and 2019, for example, about 100 articles have been indexed on PubMed alone that reported the use of a Cerulein-induced pancreatitis mouse model.

In humans, abdominal pain is the most common symptom observed in acute pancreatitis, and can range from mild to severe (Frossard et al., 2008; Banks et al., 2010). While pancreatitis can occur in many companion animal species, pain as a symptom is sometimes difficult to diagnose. Abdominal pain with acute pancreatitis is reported more often in dogs than in cats (Zoran, 2006; Xenoulis, 2015). Horses with acute pancreatitis present with severe abdominal pain (Bakos et al., 2008; Gomez et al., 2015). In translational pancreatitis research, rodents are used most frequently. According to symptoms reported by human patients, and as seen in veterinary practice, one would suspect high pain levels also in rodents. Indeed, authorities granting animal licenses in Switzerland rank the model of acute pancreatitis in mice with the highest degree of severity because experimental evidence suggests that some acute pancreatitis models are painful in mice (Michalski et al., 2007; Stumpf et al., 2016). Nevertheless, to date, there is no standardized pain assessment in these models. Michalski et al. reported that C57BL/6J mice with acute Cerulein-induced pancreatitis show a higher frequency of nocifensive reactions to von Frey stimuli of the abdomen, which could be alleviated with a synthetic analgesia-inducing cannabinoid (Michalski et al., 2007). Similarly, in a study by Stumpf et al., C57BL/6J mice with acute Cerulein-induced pancreatitis display increased abdominal hypersensitivity and neural activity in the thalamus and hypothalamus – the main brain regions involved in pain. These symptoms could be decreased with orally administered Metamizole (Stumpf et al., 2016).

In humans, severe pain occurring with acute pancreatitis is treated with narcotics like morphine or fentanyl, while milder or recovering cases are treated with non-steroidal anti-inflammatory drugs (Banks et al., 2010). Although it is assumed that, as in humans, acute pancreatitis in mice is painful, mice with induced pancreatitis are not treated in the same manner. Stumpf et al. (2016) reported that 98% of studies do not report the use of analgesia in pancreatitis mouse models. On the one hand, there might be valid scientific reasons to withhold analgesia, e.g., when studying pain induced by acute pancreatitis. On the other hand, researchers might fear the interference of analgesics with scientific readouts, e.g., potential impacts on inflammatory processes, hindering the wide use of analgesia in this model. Most obviously, the use of anti-inflammatory drugs is limited as they can reduce the induced pancreatitis (Bai et al., 2012). Studies available to date on other analgesic classes show conflicting results. Barlass et al. showed that morphine increases acute pancreatitis and reduces regeneration in mice (Barlass et al., 2018). Buprenorphine applied in rats is reported to either reduce or not influence pancreatitis (Wereszczyñska-Siemiatkowska et al., 1987; Ogden et al., 1994; Kim et al., 2004). The previously mentioned study by Stumpf et al. indicated that Metamizole does not influence acute pancreatitis in mice (Stumpf et al., 2016). In conclusion, researchers interested in investigating pancreatitis must choose an adequate analgesic substance carefully, but, at the same time, the available literature on pain management in these particular models is limited. As untreated pain can lead to side effects and influence experimental data (Jirkof, 2017), and also considering the refinement principle, there is a need to identify suitable treatment options for mice undergoing induced pancreatitis.

In the present study, we aim to evaluate pain and analyze several analgesics for their efficacy and side effects in the acute Cerulein-induced pancreatitis mouse model. We used physiological and behavioral pain-related parameters as well as parameters of general well-being to grade pain, stress, and well-being of male C57BL/6J mice with acute pancreatitis. More precisely, we measured body weight as a general parameter of health and water intake to calculate the intake of analgesics administered *via* drinking water. Burrowing behavior was tested to detect reduced well-being and impairment of the animal’s general condition occurring with painful states. The animal was observed to detect behaviors that can hint at abdominal pain, for example, the so-called “stretch-and-press” or a reduced rear up frequency. The Mouse Grimace Scale (MGS), as a general indicator of pain, as well as the von Frey test to assess abdominal hypersensitivity where the pancreas is located, were used to grade pain or hypersensitivity resulting from pancreatitis. Fecal corticosterone metabolites (FCMs) were analyzed to monitor adrenocortical activity and stress hormone release. To investigate the manifestation of pancreatitis, levels of amylase and lipase in the blood were analyzed and the pancreatic tissue was investigated histologically. To control for the impact of handling and injection procedures, a control group of NaCl-injected animals was added. Additionally, we assessed if pain can be relieved with systemic analgesia, namely Buprenorphine, a combination of Paracetamol and Tramadol, or Metamizole, administered *via* the drinking water. The impact of these analgesics on the typical histological read-out parameters used commonly in this model was part of the investigation. We hypothesize that acute Cerulein-induced pancreatitis is painful in mice and that the pain can be alleviated with common analgesics.

## Materials and Methods

### Ethics Statement

The Cantonal Veterinary Office, Zürich, Switzerland, approved animal housing and experimental procedures under license no. 097/2017. All procedures were in accordance with the Swiss Animal Protection Law and conform to European Directive 2010/63/EU of the European Parliament and of the Council on the Protection of Animals used for Scientific Purposes. The manuscript was prepared according to ARRIVE guidelines (Kilkenny et al., 2010).

### Animals

A total of 90 male C57BL/6J mice from Charles River Laboratories Sulzfeld Germany aged 8–10 weeks were used. One animal died after the first injection of Cerulein. A macroscopic post-mortem examination did not reveal any anomalies. In the last experimental session, Cerulein injections did not result in histologically detectable pancreatitis. We assume the failure occurred due to incorrect storage of Cerulein. Data from the animal that died prematurely and the seven animals receiving non-functioning Cerulein from the last session were excluded from the analysis.

### Standard Housing Conditions

Mice were accustomed to housing conditions for 2 weeks after delivery while being kept in groups of eight in Eurostandard Type III open-top, clear plastic cages with wire cover (Techniplast, Hohenpeißenberg, Germany) with 21 ± 1°C, 45 ± 5% humidity, and a light/dark cycle of 12 h/12 h (lights on at 8 am). The cages were filled with autoclaved dust-free sawdust bedding (80–90 g/cage; LTE E-001 Abedd, Indulab, Gams, Switzerland). One tissue paper, two cotton nestlets (5 cm × 5 cm, Indulab, Gams, Switzerland), and paper fibers (Enviro-dri, Shepard Specialty Papers, United States) were provided as nesting material. The cages were equipped with a cardboard hut (Ketchum Manufacturing, Brockville, Canada) and a red plastic house (Techniplast, Hohenpeißenberg, Germany). Food (Kliba Nafag no. 3436, Granovit AG, Kaiseraugst, Switzerland) in the food hopper and sterilized drinking water was provided *ad libitum*. From the start of acclimation, a clear handling tunnel was provided in each group cage (Datesand group, Stockport, United Kingdom). During the second week, animals were subjected to tunnel handling training with the familiar cage tunnel according to Hurst et al. for about 5 min per cage and day (Hurst and West, 2010). During the subsequent experiments, animals were tunnel handled only.

A health surveillance program was performed according to FELASA guidelines. The mice were free of all viral, bacterial, and parasitic pathogens listed in FELASA recommendations (Mahler Convenor et al., 2014).

### Treatment Groups

Animals were allocated randomly to one of five treatment groups, each containing 18 mice. The first group with intraperitoneal (i.p.) saline injections served as a control group (**NaCl**). Animals in the remaining experimental groups were all injected i.p. with Cerulein but differed in further treatment: (i) no further treatment (**Cer**), (ii) Buprenorphine (**Cer + Bup**), (iii) Paracetamol and Tramadol (**Cer + PT**), or (iv) Metamizole (**Cer + Met**). All analgesic drugs were administered *via* the drinking water.

### Experimental Schedule

**FIGURE 1:**
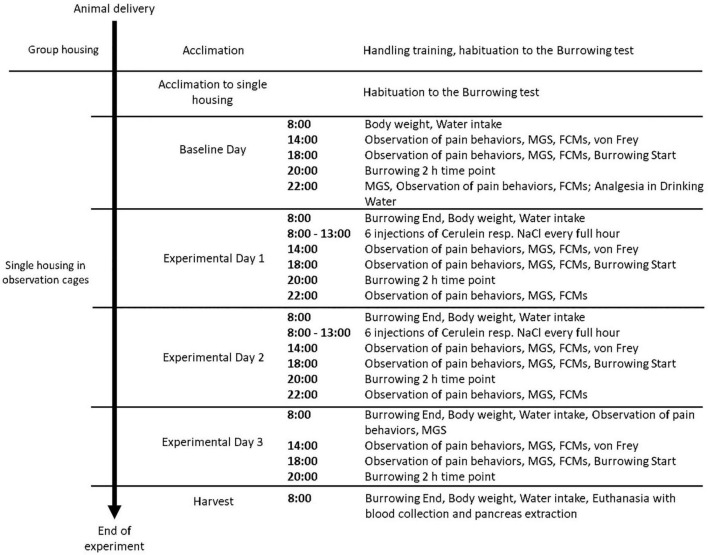
Experimental Schedule of procedures performed in each treatment group. Animals were assigned randomly to a treatment group (*n* = 18, NaCl, Cer, Cer + Bup, Cer + PT, Cer + Met). Each parameter was assessed at baseline level in naïve mice, followed by measurements conducted on Experimental Days 1–3 and the day of harvest. MGS = Mouse Grimace Scale, FCMs = Fecal Corticosterone Metabolites.

After 2 weeks of acclimation with 1-week tunnel handling training in group housing, mice were separated and housed individually in observation cages (Eurostandard Type III with usual lid, food hopper, and bedding, one cotton nestlet was provided) (Figure 1). A drinking bottle with a bent sipper including a marble to reduce spillage was attached to the outside wall (300 ml bottle, TD200 nipple, UNO BV, Netherlands). Mice were allowed to acclimate for 24 h before the subsequent baseline and experimental measurements. All experiments were conducted by female researchers. In this phase, animals were tunnel handled with an individual tunnel not present in the cage to avoid implications with the experimental setup.

At the end of the experiment, blood was withdrawn from the mouse’s heart in deep anesthesia (Sevoflurane 8%), animals were euthanized with cervical dislocation, and the pancreas was extracted carefully.

#### Intraperitoneal Injections

Pancreatitis was induced by a total of 12 i.p. Cerulein (Bachem AG, Switzerland) injections of 50 μg/kg in 0.9% NaCl, distributed on experimental days 1 and 2 (Malagola et al., 2019; Figure 1). Mice from the control group were injected with equal volumes of 0.9% NaCl.

#### Analgesia

We expected a water intake of at least 3 ml per 24 h in a healthy adult mouse and chose the analgesic dosages accordingly. For a sought dosage of 1 mg/kg for Buprenorphine, we administered 0.0094 mg/ml in the drinking water (Temgesic^®^, Schering-Plough). To reach the recommended Paracetamol dosage of 200 mg/kg, we used 4 mg/ml (Dafalgan^®^ children’s syrup, Bristol-Myers Squibb SA, Switzerland). Tramadol was added to the Paracetamol containing drinking water at a dose of 1 mg/ml to reach the sought dosage of 25 mg/kg (Tramal^®^, Grünenthal, Germany). The sought dosage of Metamizole was 200 mg/kg, therefore, 1.25 mg/ml was given in the drinking water (Novalgin^®^, Sanofi, France).

#### Body Weight and Water Intake

Body weight was measured daily by weighing animals on the precision scale. Daily water intake was measured by weighing the drinking bottles on a precision scale. Analgesia intake was calculated from the weighed daily water intake.

#### Burrowing

Burrowing behavior was tested according to Jirkof et al. (2010). Each animal was provided with a tube-like apparatus (250 ml drinking water bottle; 15 cm in length, 5.6 cm diameter on closed-end, 3 cm diameter on open-end) filled with pre-weighed food pellets (200 ± 10 g) 2 h before the beginning of the dark phase (18:00). The filled tube was weighed after 2 h (20:00) and at the end of the dark phase (8:00) to assess the weight of removed food pellets and to evaluate burrowing performance. Before the baseline experiments, animals were habituated to the test once in group housing and once on the day the mice were separated.

#### Observation of Pain Behaviors

Mice were filmed individually in transparent plastic observation boxes (10 × 10 × 14 cm) with a digital single-lens reflex camera (distance to mice 50 cm, Canon EOS 750D) for 10 min. The videos were analyzed by a blinded observer for the frequency of rear-ups in 10 min and whether pressing (abdomen pressed to the floor) and stretch-and-press (abdomen pressed to the floor while the whole body is stretched and hind legs extended) behaviors were displayed (Leach et al., 2012).

#### Mouse Grimace Scale (MGS)

The assessment of pain behaviors was followed by the assessment of the MGS. Mice were transferred to polycarbonate boxes with colorless-transparent front and back, and red-transparent walls (9 × 5 × 5 cm). These boxes were placed in a white light tent (80 × 80 × 80 cm), illuminated at an angle with two light bulbs (distance to animals 55 cm, 135 W). Mice were allowed to acclimate in the boxes for 2 min and were then filmed for 5 min with a digital single-lens reflex camera (Supplementary Figure 2, Supplementary Material). An automatic frame production and selection software produced 5 pictures per animal (Kopaczka et al., 2018), which were randomized automatically and scored by two blinded researchers according to Langford et al. (2010). The mean of the five MGS characteristics was calculated, and the means of both examiners resulted in the final MGS score. Individual picture ratings where the mean MGS differed > 0.5 between raters were rare and were discussed until agreement was reached.

#### Fecal Corticosterone Metabolites (FCMs)

At the time points at which MGS was assessed, available feces of each animal were collected from the box and stored immediately at –20°C. The fecal samples were processed according to Touma et al. and FCM concentrations were expressed in ng/0.05 g (Touma et al., 2003, 2004).

#### Von Frey Test

Hypersensitivity of the abdominal area of the pancreas to mechanical stimuli was quantified by assessing nociceptive behaviors (Takemura et al., 2011). After the MGS test, mice were put on a wire grid (mesh opening 0.3 mm) and covered by the MGS box. After an acclimation time of 3 min, a filament (strength 4, bending force 1 g) was applied to the left upper quadrant where the pancreas is located five times within 5–10 s and, after a rest of 1 min, another five times, for a total of 10 applications. Nociceptive behavior was scored for each application of the filament (0 = no response, 1 = immediate scratching/licking of the stimulated site, 2 = strong retraction of the abdomen or jumping). The resulting scores for the 10 challenges were added to give a total reported score.

#### Serum Analysis

At the end of the experiment when mice were euthanized, blood acquired was transferred to Eppendorf tubes and centrifuged (10 min, 6500 × *g*). The supernatant was collected and stored at –20°C. Amylase and lipase were determined in the serum according to Zechner et al. (2012) and results were presented in U/l.

#### Histology

The pancreas was extracted carefully after euthanasia, washed in saline, and kept in 4% formaldehyde for 24 h. After subsequent overnight processing in a tissue processor, organs were embedded in paraffin. Slices of 3 μm were stained with CD45 staining. Nine CD45 stained slices per animal were chosen randomly by a blinded researcher. The number of inflammatory cells was assessed as the percentage of CD45-positive cells by a blinded researcher with Image J software (Rueden et al., 2017). The mean percentage of CD45-positive cells in all nine slices represents the result for each animal.

#### Statistical Analysis

The sample size calculation was performed with G*Power (Faul et al., 2007). The number of animals included in each test is stated either in the text or figure legend. Statistical analysis was carried out with R (version 3.6.1) (R Core Team, 2019). Data were first analyzed using a linear mixed-effects regression model (*lmer*) from the *lme4* package to detect the influence of the factors treatment and time (Bates et al., 2015). The treatment and time of measuring (day and time point during the day) were treated as fixed effects, while the animal ID was treated as a random effect. Following the general analysis, treatment and time differences were further analyzed. Differences between the treatment groups were analyzed with pairwise *post hoc* tests using Bonferroni correction to adjust for multiple comparisons. Differences between baseline and experimental measurements taken once the injection protocol had started, were analyzed within each treatment group using a pairwise *post hoc* test with Dunnett’s correction. Degrees of freedom were approximated using the Kenward-Roger approximation. CD45 histological staining, serum lipase and amylase were analyzed with the Kruskal–Wallis test followed by *post hoc* pairwise Wilcoxon tests with Bonferroni correction. Differences were considered significant when *p* ≤ 0.05.

## Results

In the following results, we describe the influence of the factors treatment and time on the measured parameters. Subsequently, differences between treatment groups are illustrated with Bonferroni *post hoc* testing and the differences from the baseline values within each group are described with Dunnett’s *post hoc* testing. Additional graphs and exact *p*-values for significant results (comparison of treatment groups as well as comparison of baseline with experimental measurements made once animals were subjected to injections) can be found in the Supplementary material.

### Pancreatitis Histology CD45 Staining

**FIGURE 2:**
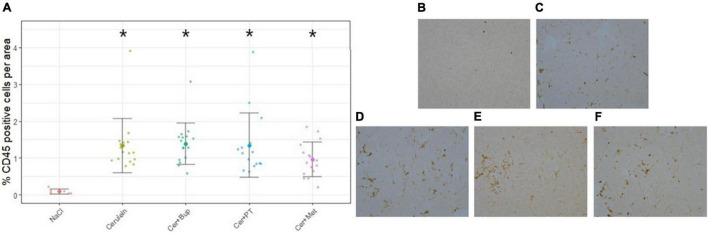
Assessment of pancreatic inflammation with CD45 immunostaining displayed as percentage of CD45 positive cells per area **(A)**. Wilcoxon *post hoc* test detected significant differences between the NaCl group and the groups receiving Cerulein. The Cerulein group and groups with Cerulein and an analgesic did not differ from each other. Examples of CD45 immunostaining of the different treatment groups: **(B)** NaCl, **(C)** Cerulein, **(D)** Cerulein with Buprenorphine, **(E)** Cerulein with Paracetamol and Tramadol and, **(F)** Cerulein with Metamizole. Data are presented as scatter dot plots with mean ± SD. *Significant (*p* ≤ 0.05) differences from NaCl treated animals. NaCl *n* = 6, Cer *n* = 17, Cer **+** Bup *n* = 16, Cer **+** PT *n* = 15, Cer **+** Met *n* = 16.

Pancreatic inflammation was assessed at the end of the experiment with CD45 immunostaining of inflammatory cells. The results are displayed as the percentage of CD45-positive cells per area for each treatment group in Figure 2. The Kruskal–Wallis test showed a significant difference between the treatment groups (chi-squared = 21.405, df = 4, *p* = 0.0003). *Post hoc* pairwise comparison with Wilcoxon rank-sum test and Bonferroni correction detected significant differences between NaCl-treated and Cerulein-treated animals (NaCl to Cerulein *p* = 0.0003; NaCl to Cer + Bup *p* = 0.0046; NaCl to Cer + PT *p* = 0.0053; NaCl to Cer + Met *p* = 0.0005). No differences were detected between the Cerulein-treated groups, regardless of whether, or which, analgesic substance was administered.

### Lipase and Amylase

At the end of the experiment, blood was withdrawn, and serum lipase and amylase were analyzed (Supplementary Figure 3). The Kruskal–Wallis test detected a significant difference in lipase between the treatment groups (chi-squared = 43.272, df = 4, *p* < 0.0001). The *post hoc* Wilcoxon rank sum test showed higher lipase in the NaCl group when compared with Cerulein (*p* < 0.0001), Cer + Bup (*p* = 0.0001), Cer + PT (*p* < 0.0001), and Cer + Met (*p* < 0.0001)-treated animals.

In serum amylase analysis, the Kruskal–Wallis test also detected a significant difference between the treatment groups (chi-squared = 30.183, df = 4, *p* < 0.0001). The *post hoc* Wilcoxon rank sum test showed higher amylase in the NaCl group when compared with the Cer + Bup (*p* < 0.001) and Cer + PT (*p* < 0.0001) groups, while the Cer + PT group had lower amylase levels than the Cerulein and Cer + Met groups (*p* < 0.01 and *p* < 0.05).

### Water and Analgesia Intake

**FIGURE 3:**
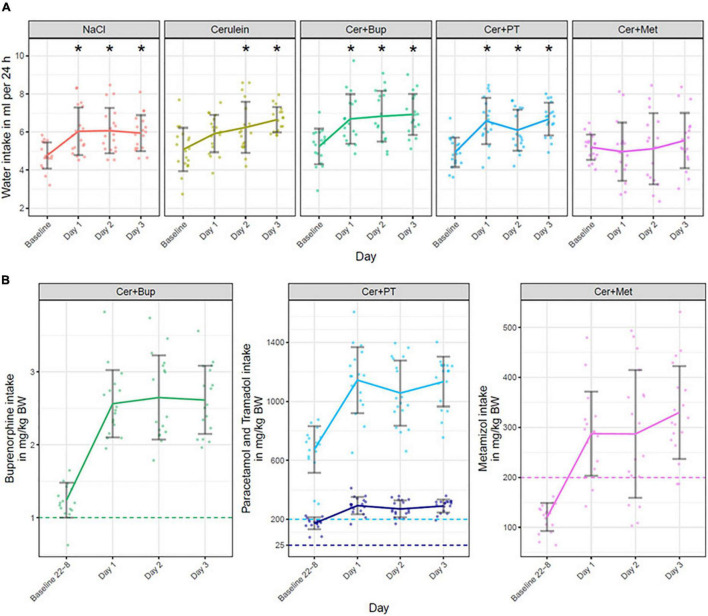
**(A)** Water intake in ml per 24 h is displayed for baseline (untreated water) and on Days 1, 2, and 3 (treated water). Bonferroni corrected *post hoc* test showed that on Day 1, Cer + Met had a significantly lower water intake than NaCl and Cer, and water intake in Cer + PT was lower than in Cer. On Day 2, water intake in Cer + Met was significantly lower than in NaCl, Cer, and Cer + Bup. On Day 3, Cer + Met had a significantly lower water intake than all other treatment groups. * Significant (*p* ≤ 0.05, Dunnett’s *post hoc*) differences to the baseline water intake within one treatment group. **(B)** Analgesia intake presented for each substance in the different treatment groups. Note that the Cer + PT group received a combination of Paracetamol (light blue) and Tramadol (dark blue). The intake is displayed in mg/kg body weight during the night before the first Cerulein injections (22:00 to 8:00, Baseline 22-8), during the 24 h of the first day of injection (Day 1), during the 24 h of the second day of injection (Day 2) and during the 24 h on the day following the injections (Day 3). The dashed line marks the dosage sought in mg/kg per 24 h. No statistical analysis was performed on the analgesia intake. Data are shown as scatter dot plot with mean ± SD, NaCl *n* = 18, Cer *n* = 17, Cer + Bup *n* = 16, Cer + PT *n* = 15, Cer + Met *n* = 16.

Water intake was measured daily by weighing water bottles to subsequently calculate analgesia intake per 24 h. The water intake is shown for each treatment group in milliliters per 24 h in Figure 3A. Baseline values in all treatment groups were measured with untreated water. With the applied linear mixed-effects regression model, we found a main effect of time (*p* < 0.05) with a significantly higher intake on the experimental days. Additionally, a main effect was found for some factor combinations, which were further investigated with *post hoc* tests.

Pairwise *post hoc* comparison of treatment groups with Bonferroni correction showed no difference in baseline intake. On Day 1, Cer + Met had a lower water intake than NaCl and Cer while Cer + PT showed a lower intake than Cer. On Day 2, Cer + Met had a significantly lower water intake compared with NaCl, Cer, and Cer + Bup. On Day 3, water intake in Cer + Met was significantly lower than in all other treatment groups.

Dunnett’s *post hoc* test detected a significantly higher water intake in NaCl, Cer + Bup, and Cer + PT groups compared with baseline on all experimental days. In Cer, water intake was significantly higher on days 2 and 3. For details, see Supplementary Tables 1, 2.

Analgesic intake is displayed in mg/kg body weight in Figure 3. The analgesics were administered in the drinking water beginning at the evening before the first Cerulein injection on Day 1. Preemptive analgesia was thus given for 10 h to reach effective blood levels of analgesics once Cerulein treatment started. In the Cer + Bup group, 15 out of 16 mice reached the desired Buprenorphine dosage of 1 mg/kg per 24 h during the 10 h of preemptive treatment. All Cer + Bup mice reached the sought Buprenorphine dosage in the days during and following injection. In the Cer + PT group, all animals reached sought dosages of Paracetamol and Tramadol over the complete treatment period also in the 10 h of preemptive treatment. In contrast, Metamizole intake was lower than the 24 h dosage sought during the first 10 h of treatment. Two animals did not reach the sought dosage of Metamizole on the day of first injections (Day 1), four did not reach it on the day of second injections (Day 2), and two animals did not reach it during the last day.

### Body Weight

**FIGURE 4:**
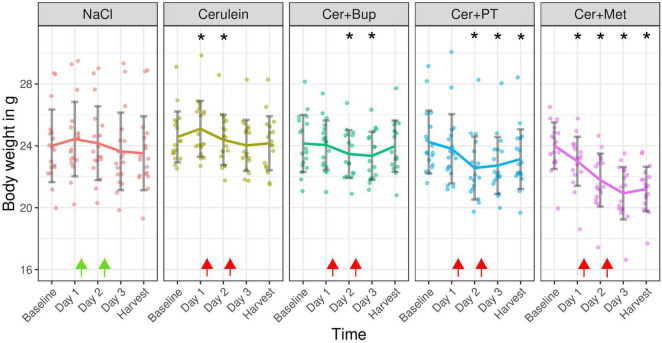
Absolute body weight in grams measured on the day before experimental treatment (Baseline), immediately before the first (Day 1) and the second (Day 2) injections, on the morning of Day 3, and on the harvest day. Arrows indicate injection time points (green = NaCl, red = Cerulein). Bonferroni corrected *post hoc* test showed a lower body weight on Day 1 inCer + Met compared with Cer. On Day 2, Cer + Met body weight was lower than NaCl and Cer. On Day 3, Cer + Met body weight was lower than NaCl, Cer, Cer + Bup. On the day of harvest, Cer + Met body weight was lower than all other groups. *Significant (*p* ≤ 0.05, Dunnett’s *post hoc*) differences to the baseline weight within one treatment group. Data are shown as scatter dot plot with mean ± SD. NaCl *n* = 18, Cer *n* = 17, Cer + Bup *n* = 16, Cer + PT *n* = 15, Cer + Met *n* = 16.

Body weight was measured daily, also on days 1 and 2 before the first injection, to assess the general well-being and health state of the animals. The results are displayed as absolute values in grams in Figure 4. With the applied linear mixed-effects regression model, we found a main effect of time with a decreased body weight from Day 2 (*p* < 0.05). Additionally, a main effect was found for some factor combinations, which were further investigated with *post hoc* tests.

Pairwise *post hoc* comparison of treatment groups with Bonferroni correction showed a significantly lower body weight in Cer + Met animals compared to Cer on the morning of Day 1. On the morning of Day 2, Cer + Met had a significantly lower body weight than NaCl and Cerulein-treated animals. On the morning of Day 3, a significantly lower body weight in Cer + Met compared to NaCl, Cer and Cer + Bup was detected. Just before the harvest, Cer + Met showed a lower body weight compared to all other treatment groups.

Dunnett’s *post hoc* test detected a significantly higher body weight in Cer on Day 1, and significantly lower body weight on Day 3 compared to baseline. Body weight in the Cer + Bup group was significantly lower than baseline on days 2 and 3. In Cer + PT, the body weight on Day 2, 3, and harvest day was significantly lower than at baseline. Cer + Met showed a significantly lower body weight on all days after baseline. For details, see Supplementary Tables 3, 4.

### Behaviors Hinting at Abdominal Pain

Established behaviors indicating abdominal pain are the pressing of the abdomen against the floor as well as the so called press-and-stretch behavior where animals press their abdomen to the floor while they stretch out their complete body with extended hind-legs (Wright-Williams et al., 2007; Jirkof et al., 2015). These behaviors were assessed blindly from 10 min video recordings. Results are displayed in Table 1 as the number of animals in each treatment group that showed these behaviors regardless of frequency and duration. No statistical analysis was done. The number of animals showing the mentioned behaviors once injections were started did not change in NaCl, Cer, Cer + PT, and Cer + Met. In Cer + Bup, more animals showed the press behavior on the experimental days.

**TABLE 1:** Number of animals exhibiting pressing or stretch-and-press-behavior at the respective time points of assessment at baseline and experimental days (14:00, 18:00, 22:00 and 8:00).

Treatment	B14	B18	B22	Day1 14	Day1 18	Day1 22	Day2 14	Day2 18	Day2 22	Day3 8	Day3 14	Day3 18
**NaCl**	0	1	1	2	7	4	2	3	3	0	1	4
**Cerulein**	0	0	0	2	3	0	0	2	1	0	0	1
**Cer + Bup**	0	0	0	12	12	12	7	10	4	5	3	3
**Cer + PT**	0	2	3	3	6	0	0	3	2	5	2	1
**Cer + Met**	0	1	2	1	2	2	1	1	1	0	1	2

*Note that press behavior in mice is a known side effect for Buprenorphine, explaining the high number of mice showing this behavior in the Cer + Bup group.*

### Mouse Grimace Scale

**FIGURE 5:**
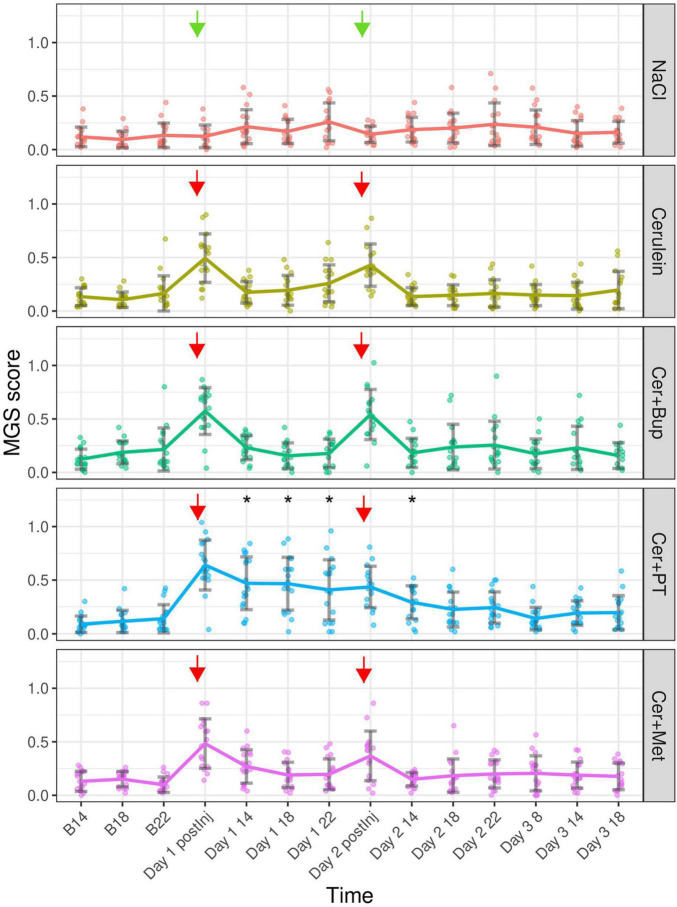
Mouse Grimace Scale scores are shown for each treatment group at baseline (B 14, B 18, and B 22), immediately after the first injection of the day (Day 1 and 2 postInj), and at different time points following the injections. Arrows marking the injection time points (green = NaCl, red = Cerulein). Bonferroni *post hoc* test detected a significantly lower MGS in NaCl compared with all other Cerulein-treated groups on days 1 and 2 immediately after the first injection. On Day 1 after the injection, Cer showed a lower MGS than Cer + Bup and Cer + PT; at the same time point on Day 2, Cer + Met showed lower scores than Cer + Bup. On Day 1 14:00, 18:00, and 22:00; and on Day 2 14, Cer + PT showed a significantly higher MGS compared with all other groups. *Significant (*p* ≤ 0.05, Dunnett’s *post hoc*) differences from the respective baseline measurements. Data are shown as scatter dot plot with mean ± SD. NaCl *n* = 18, Cer *n* = 17, Cer + Bup *n* = 16, Cer + PT *n* = 15, Cer + Met *n* = 16.

The MGS was assessed at several time points after the induction, and during the development, of pancreatitis. Baseline measurements for comparison were assessed at the respective time points before injections of NaCl/Cerulein were administered (Figure 5). The linear mixed-effects regression model showed a main effect of time, with a higher MGS on the experimental days (*p* < 0.05). Additionally, we found several main effects of factor combinations, which were further investigated with the following *post hoc* tests.

Bonferroni corrected *post hoc* group comparison detected significantly lower MGS scores in NaCl compared to the remaining four Cerulein injected groups immediately after the first Cerulein injection. Here, the Cer group showed significantly lower scores than Cer + Bup and Cer + PT. On Day 1 at 14:00, 18:00 and 22:00, meaning 1, 5, and 9 h after the last injection of the day, the MGS in Cer + PT was significantly higher than in all other groups. On Day 2, immediately after the first injection, MGS was again significantly lower in NaCl compared to the four Cerulein treated groups. At this time point, MGS in Cer + Bup was also slightly but significantly higher than Cer + Met. On Day 2 14:00, i.e., 1 h after the last injection of the day, Cer + PT showed a significantly higher MGS than Cer. At the remaining time points, Cerulein-treated groups showed no differences from NaCl treated animals, with or without analgesia.

Dunnett’s *post hoc* testing detected a significantly higher MGS in the Cer + PT group on Day 1 at 1, 5, and 9 h, and on Day 2 at 1 h after the last injection of the day compared with the corresponding baseline values. Note that there was no baseline measurement that enabled us to compare the MGS data from immediately after the first injection of the day with baseline MGS. For details, see Supplementary Tables 5, 6.

### Von Frey Test

Von Frey test was applied at baseline and then daily at 14:00 (on injection days 1 h after the last injection) to assess hypersensitivity in the abdominal region due to developing pancreatitis. The results for baseline and experimental measurements are displayed in Supplementary Figure 2, Supplementary Material. The linear mixed-effect regression model detected a main effect of time with overall higher values once treatment with NaCl/Cerulein was started (*p* < 0.05). However, *post hoc* tests did not detect a significant difference between two specific time points or between treatment groups.

### Rear Up Behavior

Rear up behavior was measured at several time points on the days of NaCl/Cerulein treatment as well as developing pancreatitis and for comparison at the respective baseline time points. The rear up frequency is presented in Supplementary Figure 3, Supplementary Material. The linear mixed-effects regression model detected a significant main effect of the factor treatment with lower values in the groups being treated with Cerulein and analgesia (*p* < 0.05). The factor time also showed significantly lower values on the experimental days (*p* < 0.05). Additionally, several main effects of factor combinations were detected, which were further investigated with subsequent *post hoc* tests.

Bonferroni corrected *post hoc* group comparison detected a significantly lower baseline frequency of rear up behavior in Cer + Met compared with NaCl and Cer at B14. At baseline 22:00, Cer + PT had a significantly lower frequency of rear up behavior compared with Cer. On Day 2 14:00, Cer + Bup showed a significantly lower frequency of rear up behavior than NaCl. On Day 3 14:00 and 18:00, Cer + PT had significantly lower frequency of rear up behavior compared with NaCl.

Dunnett’s *post hoc* testing detected a significantly lower frequency of rear up behavior in all treatment groups at 14:00 on Days 1, 2, and 3 compared with baseline. NaCl, Cer, and Cer + PT had a significantly lower frequency of rear up behavior at 18:00 on Day 1, 2, and 3 compared to baseline. In Cer + Bup, the frequency of rear up behavior was lower at 18:00 only on Days 1 and 2; in Cer + Met only on Day 3. Cer + Met showed no significant differences from baseline values at 22:00 on Day 1 and 2; all other groups had a significantly lower frequency of rear up behavior compared to baseline at that time point on Days 1 and 2. For details, see Supplementary Tables 7, 8.

### Burrowing

**FIGURE 6:**
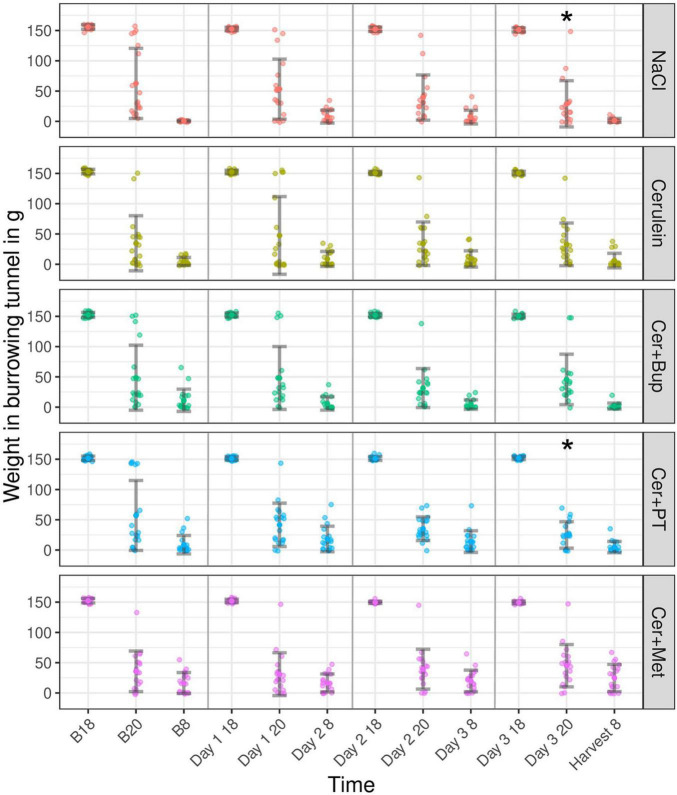
The burrowing behavior displayed as the weight in the burrowing tunnel in grams for baseline (B 18, B 20, and B 8) and experimental days (Day 1, 2, 3, and Harvest). The tunnel weight is shown at the start of the test (B18, Day 1/2/3 18), after 2 h (B 20, Day 1/2/3 20), and after 12 h (B 8, Day 2/3 8, and Harvest 8) for each treatment group. *Post hoc* testing with Bonferroni correction detected a significantly lower burrowing performance in NaCl compared to Cer and Cer + Met at B20. *Significant (*p* ≤ 0.05, Dunnett’s *post hoc*) differences to the respective baseline measurements. Data are shown as scatter dot plot with mean ± SD. NaCl *n* = 18, Cer *n* = 17, Cer + Bup *n* = 16, Cer + PT *n* = 15, Cer + Met *n* = 16.

Burrowing behavior was assessed to detect impairments in the animal’s well-being (Figure 6). With the applied linear mixed-effects regression model, we detected a main effect of time, with improving burrowing performance at 20:00 during the experiment (*p* < 0.05). At 08:00, burrowing performance was high at baseline and slightly decreased on Day 2 (*p* < 0.05). Additionally, several main effects of factor combinations were found, which were further investigated with subsequent *post hoc* tests.

Bonferroni-corrected *post hoc* testing detected significantly lower burrowing performance in the NaCl group compared with Cer and Cer + Met at B20. On the days of injection and during pancreatitis development, no group differences in burrowing performance were detected.

Dunnett’s *post hoc* testing showed that NaCl and Cer + PT had a significantly higher burrowing performance at Day 3 20:00 when compared with the respective baseline value. For details, see Supplementary Tables 9, 10.

### Fecal Corticosterone Metabolites

**FIGURE 7:**
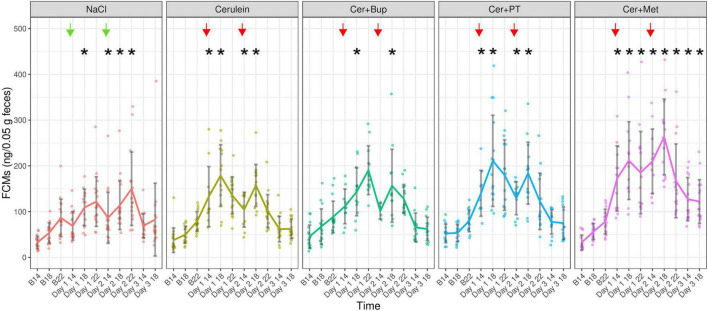
Fecal Corticosterone Metabolites (ng/0.05 g feces) were measured at the respective baseline time points (B14/18/22), and 1, 5, and 9 h after the last injection of the day, as well as at 26 and 30 h after the last overall injection. Each dot indicates the FCM concentration of one fecal sample, which can be lower than the actual animal number as feces could not be obtained from all animals. Bonferroni *post hoc* detected that on Day 1 at 14:00 and 18:00, NaCl showed significantly lower FCMs than Cer, Cer + PT, and Cer + Met while Cer + Met showed significantly higher FCMs than Cer + Bup. On Day 1 at 18:00, Cer + PT had significantly higher FCMs than Cer + Bup. On Day 1 at 22:00, significantly lower FCMs were detected in NaCl compared with the three groups with Cerulein and analgesia. On Day 2 at 14:00 and 18:00, Cer + Met treated animals showed significantly higher FCMs than the other groups and Cer + PT had significantly higher FCMs than NaCl. On Day 2 at 22:00, Cer + Met had higher FCMs than Cer. On Day 3, Cer + Met showed significantly higher FCM levels than NaCl, Cer, and Cer + Bup at 14:00. At 18:00 Cer + Met had higher FCMs than Cer and Cer + Bup. *Significant (*p* ≤ 0.05) differences from the respective baseline measurements. Data are shown as scatter dot plot with mean ± SD. NaCl *n* = 18, Cer *n* = 17, Cer + Bup *n* = 16, Cer + PT *n* = 15, Cer + Met *n* = 16.

Fecal corticosterone metabolites were assessed at several time points before and after the induction of pancreatitis and during its development from voluntarily voided feces (Figure 7). The linear mixed-effects regression model showed a main effect of time with higher concentrations on the experimental days (*p* < 0.05). Additionally, we found several main effects of factor combinations, which were further investigated with subsequent *post hoc* tests.

Bonferroni corrected *post hoc* testing detected no significant differences between the treatment groups at baseline measurements. At 1 h and 6 h after the last injection of Day 1, NaCl-injected animals had significantly lower FCMs than Cer, Cer + PT, and Cer + Met. Here, Cer + Met showed significantly higher FCMs than Cer + Bup. Additionally, on Day 1 18:00, Cer + PT also had significantly higher FCMs than Cer + Bup. At 9 h after the last injection of Day 1, significantly lower FCMs were detected in the NaCl group compared with the three groups with Cerulein and analgesia. At 1 h and 6 h after the last injection of Day 2, Cer + Met treated animals showed significantly higher FCMs than all other treatment groups. Additionally, on Day 2 18:00, Cer + PT also had significantly higher FCMs than NaCl. At 9 h after the last injection of Day 2, Cer + Met still had higher FCMs than Cer. On Day 3, the first day without injections, Cer + Met showed significantly higher FCM levels than NaCl, Cer, and Cer + Bup at 14:00. At 18:00, Cer + Met had higher FCMs than Cer and Cer + Bup.

Dunnett’s *post hoc* testing showed that NaCl injected animals had significantly higher FCMs compared with their respective baseline values on Day 1 at 18:00 and each time point on Day 2. The Cer group showed significantly higher FCMs compared to baseline on Day 1 and Day 2 at 14:00 and 18:00. Cer + Bup animals had significantly higher FCMs compared with baseline on Day 1 and Day 2 at 18:00. In Cer + PT treated animals, significantly higher FCMs compared with baseline were detected on Days 1 and 2 at 14:00 and 18:00. While the NaCl group reached baseline levels on Day 3 and Cer, Cer + Bup, and Cer + PT all reached baseline levels on Day 2 at 22:00, Cer + Met had significantly elevated FCMs compared with baseline in each of the experimental time points until the last one. For details, see Supplementary Tables 11, 12.

## Discussion

In our study, we investigated pain and analgesia in mice in a Cerulein-induced acute pancreatitis model. Although these mice had histologically verified pancreatitis, they showed no signs of pain during the severest stages of pancreatitis. However, we detected an acute increase in MGS immediately after Cerulein injections, suggesting that i.p. injection of this substance was indeed causing pain or at least discomfort. Mice with Buprenorphine or Paracetamol in combination with Tramadol in the drinking water had a sufficient calculated analgesic uptake, whereas with Metamizole some animals did not reach the target levels of analgesic uptake. Nevertheless, the analgesics used neither reduced the pain signs after Cerulein injection nor affected the pancreatic inflammation in our model.

Histological analysis of pancreatic tissue was used to verify that our Cerulein injection regime induced pancreatitis. All Cerulein-injected animals showed a significantly higher percentage of CD45-positive stained cells in the pancreas than the NaCl-treated group, suggesting that the applied protocol with 12 injections on two consecutive days induced pancreatitis in our mice. There was no difference in the percentage of CD45-stained cells between the Cerulein-treated groups regardless of whether, or which, analgesic was administered. A argument used frequently in opposition to providing pain medication in models like the pancreatitis model is the fear of affecting inflammatory processes and therefore the read-out parameters (Stumpf et al., 2016). Data on these effects is still contradictory, several papers report either negative or absent influence of analgesics (Wereszczyñska-Siemiatkowska et al., 1987; Ogden et al., 1994; Kim et al., 2004; Bai et al., 2012; Stumpf et al., 2016; Barlass et al., 2018). Moreover, the effect of the same analgesic, in this case, Rofecoxib, can differ between species (Reding et al., 2006; Silva et al., 2011). Our data indicated that Buprenorphine, Paracetamol in combination with Tramadol, or Metamizole did not influence the extent of the pancreatic inflammation in our model. With regard to the available literature and our results, it might be advisory to assess the effects of analgesic substances on the specific pancreatitis model and its read-out parameters before administration of analgesia.

High serum amylase and lipase levels are indicative of pancreatitis (Zechner et al., 2014). In contrast, we detected high levels in NaCl-treated animals which obviously did not have pancreatitis while Cerulein injected animals showed low levels (see Supplementary material). Amylase and lipase are measured routinely early after Cerulein induction in acute pancreatitis (Jurik et al., 2014; Sakuma et al., 2018); secretion of amylase and lipase in inflamed tissue is highest 8 h after the first Cerulein injection and 3 h after the last Cerulein injection, respectively. We acquired blood for enzyme analysis at the end of our experiment, 2 days after the last Cerulein injections. While this time before euthanasia allowed for behavioral testing during pancreatitis development, it also led to a belated analysis where the elevated enzymes due to pancreatitis seem to be no longer apparent. For further investigations, detailed histology should be kept in mind and could be further analyzed with immunohistochemical procedures to quantify and qualify intact acini, transdifferentiation cells, and tubular structures known as ADMs.

Water intake per 24 h was measured by weighing drinking bottles to check for sufficient analgesia uptake. The intake of untreated water in NaCl and Cerulein injected animals, as well as intake with Buprenorphine or Paracetamol in combination with Tramadol, increased during the experiment but showed rather high variability. We saw that Metamizole-treated animals had a lower water intake compared with the other groups; they also showed the highest variability in water intake, some had a much lower intake than expected for C57BL/6J mice (Bachmanov et al., 2002). This may have led to dehydration and the detected body weight loss in Metamizole-treated animals. Some of these animals did not reach the Metamizole target dose for 24 h on days 1 and 2 when the Cerulein injections took place and on Day 3 when pancreatitis was fully developing. Additionally, while with Buprenorphine or Paracetamol in combination with Tramadol, most animals reached the 24 h target dose during the first 10 h (time from 22:00 to 8:00) before the first injections on Day 1, Metamizole-treated animals did not reach the target dose in this first 10 h. Therefore, the desired preemptive analgesia was not achieved with Metamizole and, in some animals, a sufficient Metamizole uptake was also not reached during the experiment. It must be noted that we did not verify analgesic uptake by blood analysis. This would have added more procedures, potentially increasing the burden for animals and the need for additional animals. Therefore, we chose to calculate the analgesic uptake from water intake as previously described in the literature (Fleischmann et al., 2017). Although we saw some animals with a calculated intake of analgesia higher than the sought dosage per 24 h, we did not observe any signs of overdosing, which was in line with our previous work (unpublished data). Overall, we saw a high water intake per 24 h in some animals regardless of treatment. Therefore, we cannot exclude that some of the calculated water intake was indeed a result of spillage. If this was the case, the real analgesic intake might have been lower than calculated, which may have limited the risk of overdosing even further but was still enough to reach target dosages.

Body weight was assessed frequently in our study to detect impaired health and pain due to pancreatitis. The expected body weight loss in animals with Cerulein injections and pancreatitis did not occur. Indeed, body weight did not differ between the NaCl- and Cerulein-treated animals and was not reduced with frequent injections of either NaCl or Cerulein, or with developing pancreatitis. In the literature, body weight loss, as well as no effect of pancreatitis on the body weight in mice, is reported (Abdelrahman et al., 2019; Kumstel et al., 2019). The fact that we did not see weight loss in animals with untreated pancreatitis hinted at an unimpaired health of these animals in our model. In contrast, body weights decreased with analgesia in the drinking water, most severely with Metamizole, where a significant decrease was already detected on the morning of Day 1, 10 h after Metamizole was first provided with the drinking water. On the last day of the experiment, this group had a significantly lower body weight compared with all other treatment groups. We suspect that a reduced intake of Metamizole-treated water led to this decrease. Depending on the drug formulation and the manufacturer, we therefore recommend administering Metamizole in combination with a sweetener to bypass the bitter taste of Metamizole and ensure sufficient water intake.

The peak of acute Cerulein-induced pancreatitis was expected several hours after the last Cerulein injection (Lugea et al., 2006; Sakuma et al., 2018). At that time, we expected the pancreatic pain to peak, leading to an increased MGS. In contrast, we found no difference in the MGS between NaCl and Cer/Cer + Bup/Cer + Met at these time points on days 1 and 2, and until the end of the experiment. Keeping in mind that our protocol was indeed successful in inducing pancreatitis as CD45 staining indicated, this result suggests that developing pancreatitis in our model was not painful. Paracetamol + Tramadol treated animals had a higher MGS than the other groups and the respective baselines. This result was in line with our previous finding that Paracetamol increases the MGS (unpublished data). So far, we do not know the underlying cause of this.

In contrast to the low MGS at all other time points during the experiment, we detected a significantly higher MGS immediately after the first Cerulein injection on days 1 and 2 in all Cerulein injected groups, with or without analgesia. The literature on changes in MGS with pancreatitis or Cerulein injections is sparse. Jurik et al. showed a low increase of MGS 1 h after the last Cerulein injection but it is hard to define if this was caused by Cerulein or resulting pancreatitis (Jurik et al., 2014). In the study of Kumstel et al. (2019), a distress score with several parameters was used to evaluate the severity of pancreatitis. Here, only one out of four to five mice showed an abnormal posture 30 min after Cerulein injection. No other symptoms indicating pain were detected in the animals at 30 and 60 min after injection. To our knowledge, the injection of Cerulein has not been specifically reported to be painful in mice so far. Our results suggest that Cerulein, injection of which acutely increased MGS, seems to be an irritating substance that, in contrast to pancreatitis, causes pain or discomfort that could not be relieved by the analgesics used. While planning this study, our focus was on pain caused by pancreatitis. Only when the first animals were injected with Cerulein did we became aware that these animals showed changes in their facial expression indicating painful states. We therefore added an additional MGS assessment immediately after the first Cerulein injection of the day. Therefore, we did not have a baseline MGS for that time point and could not show a statistical increase from baseline for the MGS taken immediately after Cerulein injection. Nevertheless, the difference compared with NaCl-injected animals at that point clearly showed the impact of Cerulein. In order to refine this model, the negative impact of Cerulein should be reduced. The application of different analgesics should be investigated further. Another possibility for refinement could be the short-term inhalation anesthesia for the time of Cerulein injection. This would allow for nociceptive processing, ensuring an animal model as close to human pancreatitis as possible, while at the same time preventing conscious pain perception in the animal during Cerulein injection. However, inhalation anesthesia needs additional infrastructure, side effects of inhalation agents on the read-out parameters cannot be ruled out, and the advantages should be weighed against the possible negative impact of repeated anesthesia. On the other hand, one could modify the model to minimize Cerulein exposure. To induce pancreatitis, Cerulein is injected intravenously, subcutaneously, or, most commonly, intraperitoneally. As no study reported Cerulein injections to be painful so far, we do not know if subcutaneous or intravenous injections of Cerulein without direct exposure of the pancreas to Cerulein are less painful. The continuous release of Cerulein by an intraperitoneal implanted mini-osmotic pump may prevent peaks of acute, short-term pain caused by single Cerulein injections. The negative side effects of surgical implantation be considered. Additionally, researchers should evaluate if the number of Cerulein injections complies with the minimal number necessary to reach the desired read-out parameters.

We expected animals with painful acute pancreatitis to have radiating pain in the abdominal area that would be detectable as local hypersensitivity with an increased von Frey score (Takemura et al., 2011; Suzuki et al., 2012). In contrast, the von Frey score was unchanged 1 h after the last Cerulein injection of the day or with developing pancreatitis in our study. These results indicate that the accumulation of injections with either NaCl or Cerulein as well as pancreatitis itself did not induce hypersensitivity in the abdominal area in our model. It has to be noted that we did not assess von Frey immediately after Cerulein injection. There was also no difference between Cerulein-treated groups without and with analgesia, which opposes the assumption of aggravating pancreatitis with specific analgesics, as proven also by the histological analysis and the MGS.

A reduced frequency of rear up behavior as a sign of reduced well-being or pain was assessed during the 10 min of behavior observation. In our study, the rear up frequency was highly variable and rather high in all groups at baseline. It then declined during the experiment. There were no significant differences between NaCl- and Cerulein-injected animals, suggesting that the mentioned decrease in rear up frequency was not due to painful pancreatitis. We suggest that animals habituated to the testing area and thus their explorative behavior, and, with it, the rear up frequency, decreased steadily. The remaining behaviors indicative of pain in mice were shown slightly more often on experimental days than at baseline in all treatment groups. Here, the NaCl, Cer, Cer + PT, and Cer + Met groups were comparable, while Cer + Bup had a higher occurrence of mainly pressing behavior. The high frequency of pressing behavior in Buprenorphine-treated mice is a known side effect of the analgesic (Jirkof et al., 2015) and does not hint at pancreatitis pain in these mice.

Burrowing behavior is used to assess changes in an animal’s well-being (Jirkof et al., 2010). Abdelrahman et al. stated that mice in the early phase of induced chronic pancreatitis were in distress as, among other parameters, burrowing performance was negatively impacted (Abdelrahman et al., 2019). In this early phase, animals were injected with three Cerulein injections each on two different days, resulting in a pancreatic inflammation that would be comparable to our acute pancreatitis. In contrast to these findings, there was no decrease in burrowing performance and no significant difference between animals with or without pancreatitis in our study. These findings were in line with the remaining pain parameters investigated so far and suggest that well-being is not significantly impaired due to pancreatitis or repeated injections in our model.

As a measurement of stress caused by several components of the pancreatitis model, FCMs were measured. To a varying extent, FCM concentrations increased during the experiment in all treatment groups, but went back to baseline values equally in NaCl, Cer, Cer + Bup, and Cer + PT. Only in Cer + Met were FCMs significantly higher than baseline until the end of the experiment and significantly higher compared with other groups. This might have been due to increased stress caused by the reduced water intake and body weight loss resulting from aversion to Metamizole in the drinking water. NaCl-treated animals had significantly lower FCMs compared with Cerulein-treated groups only 1 and 6 h after the last injection on Day 1. After this time point, FCM levels did not differ in NaCl and Cerulein injected groups. These findings suggest that the increase in FCMs was not a result of increased stress due to painful pancreatitis, but rather due to the frequent handling and injections (Balcombe et al., 2004; Jirkof et al., 2019). The initial difference in FCMs in NaCl- and Cerulein-injected animals might have been caused by the painful Cerulein injections. Abdelrahman et al. report a significant increase of FCMs after three Cerulein injections each on two days. As FCMs approached baseline levels with developing chronic pancreatitis, and no saline control was used in the study by Abdelrahman et al., it is hard to distinguish between the effects of pancreatitis versus Cerulein injections on the FCMs of these animals (Abdelrahman et al., 2019). Kumstel et al. (2019) detected a significant increase in serum corticosterone after Cerulein injection. This increase was detected before the measurement of lipase indicated pancreatitis, suggesting the Cerulein injection, rather than the developing pancreatitis, was causing a stress reaction. We therefore assume that the increase in FCMs in our study was indeed caused by the Cerulein injections. This would be in line with the increased MGS, hinting at acute pain immediately after Cerulein injection.

It has to be noted that our results may be valid only for male C57BL/6J mice. Strain and sex differences in pain and inflammation may lead to different results. As C57BL/6J male mice are used commonly for translational pancreatitis research, we focused on these. Researchers conducting pancreatitis research in mice have to consider that the presented findings may be limited to the specific Cerulein-induced acute pancreatitis model that we used. To draw general conclusions on pain and analgesia in pancreatitis in mice, different pancreatitis models should be subjected to a standardized severity assessment. Our study may give an orientation for future studies investigating severity in pancreatitis models and can provide researchers with valuable information on critical points of the animal model when creating score sheets and prospective severity assessment. Future studies should further investigate our main finding, that Cerulein injections are acutely painful. So far, we could not answer whether pain caused by Cerulein injections changes with the number of injections throughout one day. Some injection protocols might be less painful than others. With the objective of refinement in animal experiments, researchers should be encouraged to establish protocols less burdening for the mice. Additionally, as the applied analgesics Buprenorphine, Paracetamol in combination with Tramadol and Metamizol were not effective in reducing the acute pain resulting from Cerulein injections, future studies should focus on testing different analgesic substances or local anesthesia as well as treatment schemes to find effective analgesics that at the same time do not impact the scientific read-out parameters.

## Conclusion

Taken together, the results obtained could not confirm the hypothesis of our study. The assessed acute Cerulein-induced pancreatitis did not evoke signs of pain in male C57BL/6J mice. Nevertheless, we found evidence that i.p. injection of Cerulein was acutely painful, causing pain that could not be alleviated by the administered analgesics Buprenorphine, Paracetamol in combination with Tramadol, or Metamizole. We did not detect an influence of the analgesics used in our histological analysis. While the uptake of Buprenorphine, and Paracetamol in combination with Tramadol *via* the drinking water was variable, but reached sought dosage levels, the intake of Metamizole-enriched water was reduced and the target dosage was not reached reliably. In general, our study adds information to the existing but contradictory literature on the severity of pancreatitis models and the influence of analgesic on these models. Other pancreatitis models, differing in mouse strain or sex, induction protocol or duration, and of course severity, might indeed be painful and render pain management obligatory. This should be further investigated with regard to the influence of analgesics on read-out parameters in pancreatitis research. Additionally, the severity grading of Cerulein-induced pancreatitis might have to be reconsidered by researchers and authorities according to the number of Cerulein injections rather than pancreatitis itself, as our data indicates that injection of this substance is acutely painful.

## Data Availability Statement

The authors declare that all data supporting the findings of this study are available within the article and its Supplementary Material. Further information is made available by the authors upon request.

## Ethics Statement

The animal study was reviewed and approved by Cantonal Veterinary Office, Zurich, Switzerland.

## Author Contributions

MD, PJ, MK, RG, TG, and MA: study design. MD and PJ: mouse experiments and drafting manuscript. All authors: data analysis, interpretation, revising manuscript, and approved final version of the manuscript.

## Conflict of Interest

The authors declare that the research was conducted in the absence of any commercial or financial relationships that could be construed as a potential conflict of interest.

## Publisher’s Note

All claims expressed in this article are solely those of the authors and do not necessarily represent those of their affiliated organizations, or those of the publisher, the editors and the reviewers. Any product that may be evaluated in this article, or claim that may be made by its manufacturer, is not guaranteed or endorsed by the publisher.
