# Supplementary material for: Analysis of Pain and Analgesia Protocols in Acute Cerulein-Induced Pancreatitis in Male C57BL/6 Mice
Source: Front Physiol. 2021 Nov 22;12:744638. doi: 10.3389/fphys.2021.744638 (PMC8645955; doi:10.3389/fphys.2021.744638)
Supplement: Supplementary file 1 [file Data_Sheet_1.pdf]

Supplementary Material

The supplemental material contains exact p-values for significant results from Bonferroni-corrected *post hoc* comparison of treatment groups and Dunnett's *post hoc* test comparing baseline with measurements made once NaCl/Cerulein treatment was started, along with graphs and results excluded from the main manuscript.

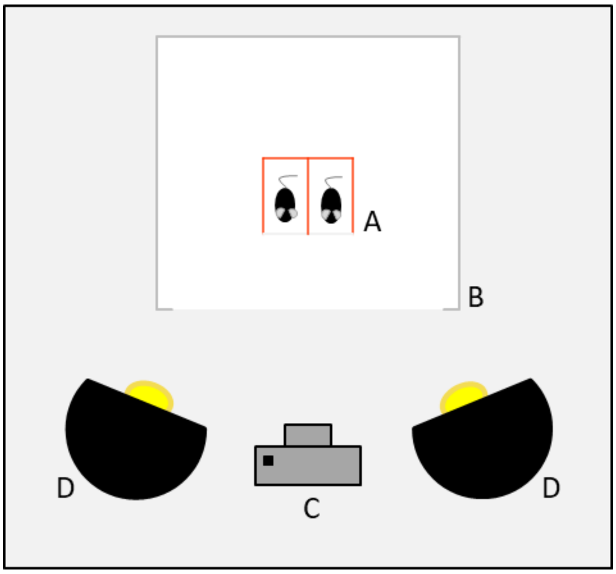

Figure S1: Schematic display of Mouse grimace scale (MGS) setup. Mice were placed in polycarbonate boxes (A) within a light tent (B). They were filmed for 5 min using a digital camera (C) while being illuminated by two light sources (D).

Water Intake

Supplementary Table S1: Bonferroni-corrected *post hoc* comparison of treatment groups for each sex and measurement time point of water intake.

Time	Contrasts	β	SE	t-value	p-value
**Day 1**	**NaCl – Cer+Met**	2.38333	0.626	3.805	0.0025
	**Cer – Cer+PT**	1.82000	0.626	2.906	0.0457
	**Cer – Cer+Met**	2.60222	0.626	4.154	0.0007
**Day 2**	**NaCl – Cer+Met**	2.70889	0.626	4.325	0.0004
	**Cer – Cer+Met**	3.09444	0.626	4.940	<0.0001
	**Cer+Bup – Cer+Met**	2.38810	0.628	3.806	0.0025
**Day3**	**NaCl – Cer+Met**	2.31444	0.626	3.695	0.0037
	**Cer – Cer+Met**	2.96000	0.626	4.726	0.0001
	**Cer+Bup – Cer+Met**	2.74398	0.628	4.373	0.0003
	**Cer+PT – Cer+Met**	1.93444	0.626	3.088	0.265

Supplementary Table S2: Dunnett's *post hoc* test of water intake comparing baseline with their respective post-procedure measurement within each treatment group.

Treatment	Contrast: Baseline to	β	SE	t-value	p-value
NaCl	Day 1	1.268	0.334	3.801	0.0027
	Day 2	1.300	0.334	3.896	0.0019
	Day 3	1.176	0.334	3.523	0.0076
Cer	Day 1	1.163	0.334	3.485	0.0087
	Day 3	1.569	0.334	4.704	0.0001
Cer+Bup	Day 1	1.462	0.340	4.303	0.0004
	Day 2	1.601	0.340	4.711	0.0001
	Day 3	1.696	0.340	4.990	<0.0001
Cer+PT	Day 1	1.643	0.334	4.925	<0.0001
	Day 2	1.162	0.334	3.483	0.0088
	Day 3	1.744	0.334	5.227	<0.0001

Body Weight

Supplementary Table S3: Bonferroni-corrected *post hoc* comparison of treatment groups for each sex and measurement time point of body weight.

Time	Contrasts	β	SE	t-value	p-value
Day 1	Cer - Cer+Met	2.09889	0.631	3.324	0.0126
Day 2	NaCl – Cer+Met	2.38333	0.631	3.775	0.0028
	Cer – Cer+PT	1.82000	0.631	2.882	0.0489
	Cer – Cer+Met	2.60222	0.631	4.121	0.0008
Day 3	NaCl – Cer+Met	2.70889	0.631	4.290	0.0004
	Cer – Cer+Met	3.09444	0.631	4.901	<0.0001
	Cer+Bup – Cer+Met	2.40990	0.632	3.811	0.0025
Harvest	NaCl – Cer+Met	2.31444	0.631	3.665	0.0041
	Cer – Cer+Met	2.96000	0.631	4.688	0.0001
	Cer+Bup – Cer+Met	2.76578	0.632	4.374	0.0003
	Cer+PT – Cer+Met	1.93444	0.631	3.064	0.0285

Supplementary Table S4: Dunnett's *post hoc* test of body weight comparing baseline with respective post-procedure measurement within each treatment group.

Treatment	Contrast: Baseline to	β	SE	t-value	p-value
Cer	Day 1	0.526	0.161	3.265	0.0242
	Day 3	-0.547	0.161	-3.392	0.0155
Cer+Bup	Day 2	-0.695	0.165	-4.220	0.0006
	Day 3	-0.811	0.165	-4.924	<0.0001
Cer+PT	Day 2	-1.680	0.161	-10.425	<0.0001
	Day 3	-1.516	0.161	-9.408	<0.0001
	Harvest	-1.109	0.161	-6.881	<0.0001
Cer+Met	Day 1	-1.008	0.161	-6.257	<0.0001
	Day 2	-2.226	0.161	-13.811	<0.0001
	Day 3	-3.077	0.161	-13.811	<0.0001
	Harvest	-2.807	0.161	-19.093	<0.0001

Mouse Grimace Scale (MGS)

Supplementary Table S5: Bonferroni-corrected *post hoc* comparison of treatment groups for each sex and measurement time point of the MGS.

Time	Contrasts	β	SE	t-value	p-value
**Day 1 immediately post injection**	**NaCl - Cer**	-0.371445	0.0538	-6.907	<0.0001
	**NaCl – Cer+Bup**	-0.460660	0.0538	-8.566	<0.0001
	**NaCl – Cer+PT**	-0.519017	0.0532	-9.758	<0.0001
	**NaCl – Cer+Met**	-0.365887	0.0544	-6.724	<0.0001
	**Cer – Cer+PT**	-0.147572	0.0518	-2.851	0.0449
	**Cer+PT – Cer+Met**	0.153130	0.0524	2.921	0.0361
**Day 1 14:00**	**NaCl – Cer+PT**	-0.256204	0.0511	-5.009	<0.0001
	**Cer – Cer+PT**	-0.295833	0.0511	-5.784	<0.0001
	**Cer+Bup – Cer+PT**	-0.244240	0.0518	-4.716	<0.0001
	**Cer+PT – Cer+Met**	0.201204	0.0511	3.934	0.0009
**Day 1 18:00**	**NaCl – Cer+PT**	-0.298554	0.0532	-5.613	<0.0001
	**Cer – Cer+PT**	-0.274088	0.0524	-5.228	<0.0001
	**Cer+Bup – Cer+PT**	-0.307348	0.0525	-5.858	<0.0001
	**Cer+PT – Cer+Met**	0.277439	0.0518	5.360	<0.0001
**Day 1 22:00**	**NaCl – Cer+PT**	-0.152455	0.0524	-3.549	0.0042
	**Cer – Cer+PT**	-0.147645	0.0518	-3.703	0.0023
	**Cer+Bup – Cer+PT**	-0.236958	0.0525	-4.982	<0.0001
	**Cer+PT – Cer+Met**	0.205571	0.0518	4.369	0.0001
**Day 2 immediately post injection**	**NaCl - Cer**	-0.286964	0.0552	-5.195	<0.0001
	**NaCl – Cer+Bup**	-0.402915	0.0553	-7.289	<0.0001
	**NaCl – Cer+PT**	-0.277838	0.0552	-5.031	<0.0001
	**NaCl – Cer+Met**	-0.222369	0.0552	-4.026	0.0006
	**Cer+Bup – Cer+Met**	0.180545	0.0537	3.361	0.0082
**Day 2 14:00**	**Cer – Cer+PT**	-0.157778	0.0511	-3.085	0.0213

Supplementary Table S6: Dunnett's *post hoc* test of the MGS comparing baseline with their respective post-procedure measurements within each treatment group.

Treatment	Contrast: Baseline to	β	SE	t-value	p-value
**Cer+PT**	**Day 1 14:00**	0.382037	0.0444	8.598	<0.0001
	**Day 1 18:00**	0.347675	0.0451	7.705	<0.0001
	**Day 1 22:00**	0.26917	0.0444	6.058	<0.0001
	**Day 2 14:00**	0.204167	0.0444	4.595	0.0003

Von Frey Test

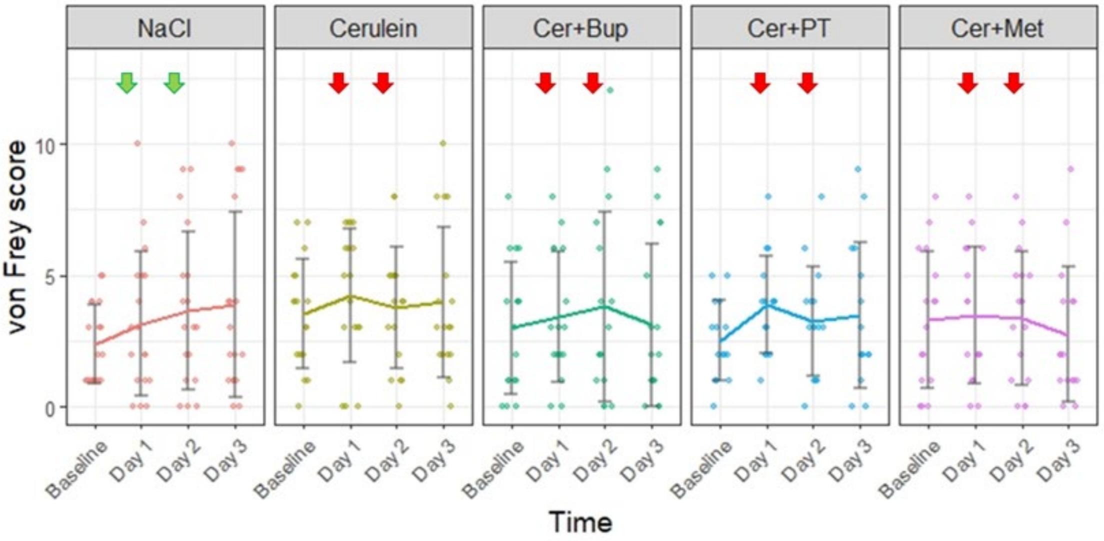

Figure S2: Von Frey scores displayed for each treatment group. The score was assessed at 14:00 without experimental treatment (Baseline), 1 h after the injections on Day 1 and 2 (Day 1, Day 2), and on the first day without injections (Day 3). Arrows marking the injection time points (green=NaCl, red=Cerulein). Bonferroni *post hoc* test detected no significant differences between the treatment groups. Dunnett's *post hoc* detected no significant differences from baseline values. Data are shown as scatter dot plots with mean ± SD. NaCl *n*=18, Cer *n*=17, Cer+Bup *n*=16, Cer+PT *n*=15, Cer+Met *n*=16.

Rear Up Frequency

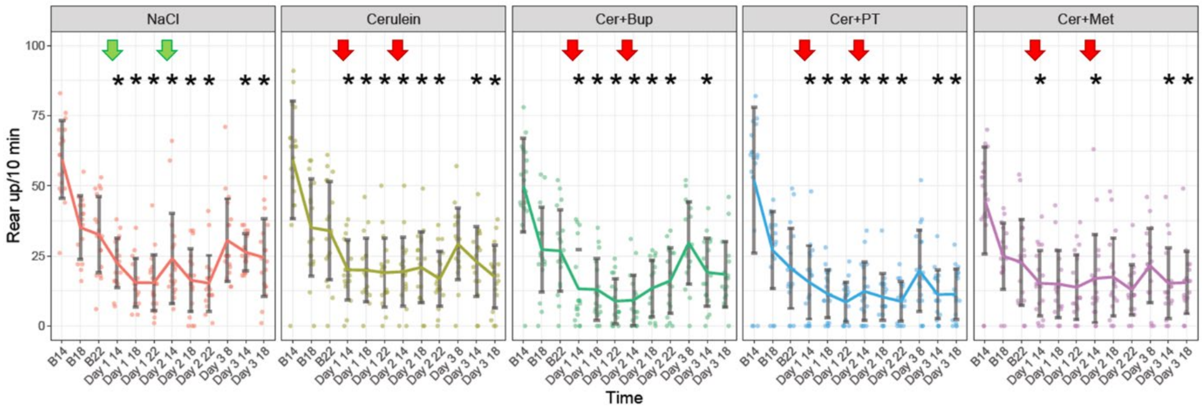

Figure S3: Rear up frequency for 10 min in each treatment group displayed for the baseline and experimental time points. Arrows marking the injection time points (green=NaCl, red=Cerulein). *Post hoc* testing with Bonferroni correction detected a significantly lower rear up frequency in Cer+Met compared with Cer and NaCl at B14. Cer+PT was significantly lower compared to Cer at B22. NaCl had a significantly higher rear up frequency compared to Cer+Bup on Day 2 14 and compared to Cer+PT on Day 3 14:00 and 18:00. *Significant ($p \leq 0.05$, Dunnett's *post hoc*) differences from the respective baseline measurements. Data are shown as scatter dot plot with mean ± SD. NaCl *n*=18, Cer *n*=17, Cer+Bup *n*=16, Cer+PT *n*=15, Cer+Met *n*=16.

Supplementary Table S7: Bonferroni-corrected *post hoc* comparison of treatment groups for each sex and measurement time point for rear up frequency.

Time	Contrasts	β	SE	t-value	p-value
**Baseline 14:00**	**NaCl – Cer+Met**	14.556	4.31	3.373	0.0085
	**Cer – Cer+Met**	14.389	4.31	3.335	0.0097
**Baseline 22:00**	**Cer – Cer+PT**	13.389	4.31	3.103	0.0211
**Day 2 14:00**	**NaCl – Cer+Bup**	15.075	4.36	3.462	0.0062
**Day 3 14:00**	**NaCl – Cer+PT**	15.111	4.31	3.502	0.0054
**Day 3 18:00**	**NaCl – Cer+PT**	13.056	4.31	3.026	0.0271

Supplementary Table S8: Dunnett's *post hoc* test of rear up frequency comparing baseline with the respective post-procedure measurement within each treatment group.

Treatment	Contrast: Baseline to	β	SE	t-value	p-value
**NaCl**	**Day 1 14:00**	-36.9	3.08	-12.003	<0.0001
	**Day 1 18:00**	-19.600	3.37	-5.813	<0.0001
	**Day 1 22:00**	-17.222	3.08	-5.596	<0.0001
	**Day 2 14:00**	-35.3	3.08	-11.480	<0.0001
	**Day 2 18:00**	-20.304	3.24	-6.264	<0.0001
	**Day 2 22:00**	-17.389	3.08	-5.650	<0.0001
	**Day 3 14:00**	-33.1	3.08	-10.758	<0.0001
	**Day 3 18:00**	-12.248	3.24	-3.779	0.0092
**Cer**	**Day 1 14:00**	-39.2	3.08	-12.744	<0.0001
	**Day 1 18:00**	-15.176	3.17	-4.792	0.0001
	**Day 1 22:00**	-14.944	3.08	-4.856	0.0001
	**Day 2 14:00**	-39.9	3.08	-12.960	<0.0001
	**Day 2 18:00**	-14.581	3.13	-4.663	0.0002
	**Day 2 22:00**	-17.167	3.08	-5.578	<0.0001
	**Day 3 14:00**	-36.3	3.08	-11.787	<0.0001
	**Day 3 18:00**	-17.970	3.13	-5.747	<0.0001
**Cer+Bup**	**Day 1 14:00**	-37.1	3.13	-11.829	<0.0001
	**Day 1 18:00**	-14.424	3.23	-4.468	0.0005
	**Day 1 22:00**	-18.150	3.13	-5.792	<0.0001
	**Day 2 14:00**	-41.1	3.13	-13.124	<0.0001
	**Day 2 18:00**	-14.424	3.18	-4.504	0.0004
	**Day 2 22:00**	-10.856	3.13	-3.464	0.0306
	**Day 3 14:00**	31.2	3.13	-9.952	<0.0001
**Cer+PT**	**Day 1 14:00**	-36.4	3.08	-11.823	<0.0001
	**Day 1 18:00**	-15.611	3.08	-5.072	<0.0001
	**Day 1 22:00**	-12.056	3.08	-3.917	0.0053
	**Day 2 14:00**	-39.6	3.08	-12.870	<0.0001
	**Day 2 18:00**	-16.722	3.08	-5.433	<0.0001
	**Day 2 22:00**	-11.833	3.08	-3.845	0.0071
	**Day 3 14:00**	-40.8	3.08	-13.249	<0.0001
	**Day 3 18:00**	-15.722	3.08	-5.108	<0.0001
**Cer+Met**	**Day 1 14:00**	-29.6	3.08	-9.621	<0.0001
	**Day 2 14:00**	-27.9	3.08	-9.061	<0.0001
	**Day 3 14:00**	-29.6	3.08	-9.621	<0.0001
	**Day 3 18:00**	-10.915	3.13	-3.491	0.0278

Burrowing

Supplementary Table S9: Bonferroni-corrected *post hoc* comparison of treatment groups for burrowing behavior.

Time	Contrasts	β	SE	t-value	p-value
Baseline 20:00	NaCl – Cer	27.98111	8.97	3.120	0.0188
	NaCl – Cer+Met	26.86167	8.97	2.995	0.0284

Supplementary Table S10: Dunnett's *post hoc* test of burrowing behavior comparing baseline with their respective post-procedure measurements within each treatment group.

Treatment	Contrast: Baseline to	β	SE	t-value	p-value
NaCl	Day 3 20:00	-33.724	9.18	-3.672	0.0092
Cer+PT	Day 3 20:00	-32.203	9.18	-3.506	0.0171

Fecal Corticosterone Metabolites (FCMs)

Supplementary Table S11: Bonferroni-corrected *post hoc* comparison of treatment groups for each sex and measurement time point of FCMs.

Time	Contrasts	β	SE	t-value	p-value
Day 1 14:00	NaCl – Cer	-59.0889	18.7	-3.166	0.0164
	NaCl – Cer+PT	-72.4054	19.8	-3.661	0.0028
	NaCl – Cer+Met	-107.4726	18.0	-5.973	<0.0001
	Cer+Bup – Cer+Met	-60.9800	20.3	-3.005	0.0278
Day 1 18:00	NaCl – Cer	-74.0452	18.8	-3.947	0.0009
	NaCl – Cer+PT	-107.6685	18.4	-5.866	<0.0001
	NaCl – Cer+Met	-104.8907	18.3	-5.736	<0.0001
	Cer+Bup – Cer+PT	-66.0822	18.7	-3.529	0.0046
	Cer+Bup – Cer+Met	-63.3043	18.7	-3.393	0.0075
Day 1 22:00	NaCl – Cer+Bup	-66.5842	18.3	-3.637	0.0030
	NaCl – Cer+PT	-58.0000	17.5	-3.312	0.0101
	NaCl – Cer+Met	-63.2875	17.2	-3.677	0.0027
Day 2 14:00	NaCl – Cer+Met	-126.0764	18.9	-6.678	<0.0001
	Cer – Cer+Met	-108.9385	20.9	-5.211	<0.0001
	Cer+Bup – Cer+Met	-114.9351	26.9	-4.272	0.0002
	Cer+PT – Cer+Met	-87.9617	20.6	-4.270	0.0002
Day 2 18:00	NaCl – Cer+PT	-70.3067	17.5	-4.015	0.0007
	NaCl – Cer+Met	-148.0959	17.7	-8.367	<0.0001
	Cer – Cer+Met	-101.6931	18.3	-5.544	<0.0001
	Cer+Bup – Cer+Met	-106.1341	19.2	-5.520	<0.0001
	Cer+PT – Cer+Met	-77.7892	18.5	-4.211	0.0003
Day 2 22:00	Cer – Cer+Met	-66.0208	17.9	-3.682	0.0026
Day 3 14:00	NaCl – Cer+Met	-56.9282	17.4	-3.264	0.0119
	Cer – Cer+Met	-64.6252	17.9	-3.616	0.0033
	Cer+Bup – Cer+Met	.62.5115	18.4	-3.402	0.0073
Day 3 18:00	Cer – Cer+Met	-59.8173	17.4	-3.428	0.0067
	Cer+Bup – Cer+Met	-61.4380	17.9	-3.432	0.0066

Supplementary Table S12: Dunnett's *post hoc* test of FCMs comparing baseline with their respective post-procedure measurements within each treatment group.

Treatment	Contrast: Baseline to	β	SE	t-value	p-value
**NaCl**	**Day 1 18:00**	53.501	15.6	3.436	0.0314
	**Day 2 14:00**	53.803	14.2	3.797	0.0080
	**Day 2 18:00**	63.816	14.7	4.344	0.0008
	**Day 2 22:00**	63.388	14.6	4.333	0.0008
**Cer**	**Day 1 14:00**	90.167	16.3	5.515	<0.0001
	**Day 1 18:00**	128.130	15.7	8.162	<0.0001
	**Day 1 22:00**	57.914	14.6	3.967	0.0040
	**Day 2 14:00**	65.897	16.9	3.906	0.0052
	**Day 2 18:00**	110.803	15.1	7.334	<0.0001
**Cer+Bup**	**Day 1 14:00**	69.630	16.9	4.118	0.0021
	**Day 1 18:00**	77.712	15.7	4.940	<0.0001
	**Day 1 22:00**	100.002	15.9	6.273	<0.0001
	**Day 2 18:00**	88.403	16.2	5.459	<0.0001
**Cer+PT**	**Day 1 14:00**	88.805	17.8	4.980	<0.0001
	**Day 1 18:00**	157.747	15.3	10.324	<0.0001
	**Day 1 22:00**	99.960	15.3	6.542	<0.0001
	**Day 2 14:00**	72.195	16.7	4.317	0.0009
	**Day 2 18:00**	130.700	15.3	8.554	<0.0001
**Cer+Met**	**Day 1 14:00**	143.633	15.7	9.150	<0.0001
	**Day 1 18:00**	155.093	15.3	10.150	<0.0001
	**Day 1 22:00**	106.713	15.1	7.086	<0.0001
	**Day 2 14:00**	179.917	16.5	10.891	<0.0001
	**Day 2 18:00**	208.614	15.6	13.351	<0.0001
	**Day 2 22:00**	88.887	15.6	5.690	<0.0001
	**Day 3 14:00**	93.427	15.1	6.203	<0.0001
	**Day 3 18:00**	68.878	15.1	4.573	0.0003

Lipase and Amylase

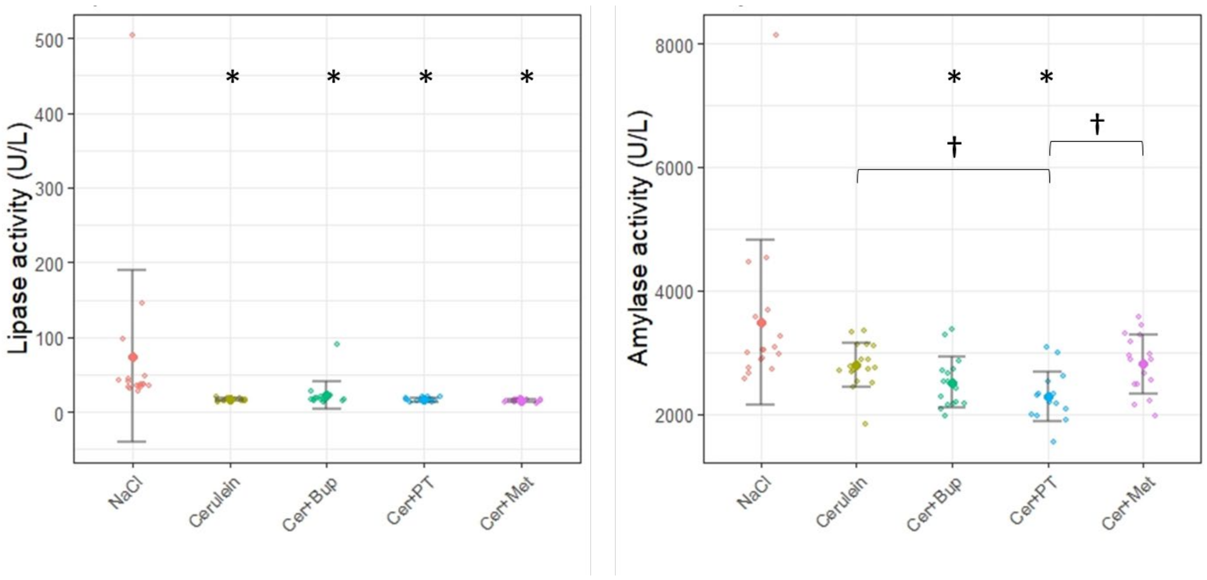

Figure S4: Lipase and amylase (U/L) in each treatment group measured on harvest day. Data are shown as scatter dot plots with mean ± SD. *Significant (p≤0.05, *post hoc* Wilcoxon rank sum test) differences from NaCl-treated animals. †Significant (p<0.05) differences between treatment groups. NaCl *n*=18, Cer *n*=17, Cer+Bup *n*=16, Cer+PT *n*=15, Cer+Met *n*=16.
